# Abnormal Heart Rate Recovery and Chronotropic Incompetence With Exercise in Patients With Interstitial Lung Disease With and Without Pulmonary Hypertension

**DOI:** 10.7759/cureus.60056

**Published:** 2024-05-10

**Authors:** Viral Vaddoriya, Sara Z Khan, Joseph L Simonson, Rammohan Gumpeni, Arunabh Talwar

**Affiliations:** 1 Department of Pulmonary, Critical Care and Sleep Medicine, Northwell Health, New Hyde Park, USA; 2 Department of Medicine, Berkshire Medical Center, Pittsfield, USA; 3 Department of Pulmonary Medicine, NewYork-Presbyterian Queens Hospital, Flushing, USA

**Keywords:** interstitial lung disease with pulmonary hypertension (ild-phtn), interstitial lung disease (ild), heart rate recovery at 1-minute post-exercise (hrr1), chronotropic response index (cri), autonomic dysfunction, chronotropic incompetence

## Abstract

Introduction

Chronotropic incompetence (CI) and heart rate (HR) recovery at one minute post-exercise (HRR1) have been proposed as indicators of autonomic imbalance. We retrospectively studied the presence of CI and HRR1 attained on cardiopulmonary exercise testing (CPET) in patients with interstitial lung disease (ILD) and those with interstitial lung disease with pulmonary hypertension (ILD-PHTN).

Methods

A total of 32 patients (21 had ILD alone; 11 had ILD-PHTN) underwent CPET performed per American Thoracic Society protocol on a manually-braked bicycle. HRR1 was defined as the difference between peak HR and HR after one minute post-exercise. The utilization of HR reserve recovery at peak exercise was expressed as Chronotropic Response Index (CRI) and was calculated as (peak HR-resting HR)/(220-age-resting HR). CI was defined by failure to reach 85% of the age-predicted maximum heart rate (APMHR = 200-Age) and CRI<0.80 (80%).

Results

VO_2_max was lower in patients with ILD-PHTN compared to ILD alone (14.15± 5.00 vs. 18.11± 4.48, *p*<0.05). Mean CRI (0.468± 0.179 versus 0.691± 0.210, *p*<0.05) and HRR1 (10± 7 versus 18± 9, *p*<0.05) were lower in patients with ILD-PHTN compared to ILD alone. Twenty out of a total of 32 patients (62.5%) met the criteria for CI. In the ILD group, 10 out of 21 patients (47.62%) and in the ILD-PHTN group 10 of 11 patients (90.90%) had CI.

Conclusion

Chronotropic Incompetence and abnormal heart rate recovery at one minute post-exercise are notable in patients with ILD and are more severe in patients with ILD-PHTN. These findings may contribute to our understanding of dyspnea due to these conditions.

## Introduction

Interstitial lung disease (ILD) and pulmonary hypertension result in significant morbidity, mortality and impaired quality of life [1]. Dyspnea in these conditions is multifactorial in origin. Cardiopulmonary exercise testing (CPET) plays a vital role in the comprehensive evaluation of patients afflicted with advanced lung diseases [2-4]. It is well established that progressive increase in heart rate (HR) is a hallmark of normal response to exercise [2,5]. Chronotropic incompetence (CI) is generally defined as the inability to increase the heart rate adequately during exercise to match the cardiac output to the metabolic demands [6]. CI has been demonstrated in patients with cardiac conditions [7] as well as in patients with chronic pulmonary obstructive diseases [6,8,9]. It has also been proposed as a marker of mortality in patients with congestive heart failure and cardiomyopathy [7,10,11].

It is also recognized that increase in heart rate with exercise is finely controlled by the autonomic system; particularly there is an increase in sympathetic activity and parasympathetic withdrawal. On the other hand, recovery of heart rate during the initial resting period after exercise is a function of parasympathetic reactivity [12]. A slower one-minute rapid decline in heart rate following exercise is also a marker of increased mortality [13-15]. Thus, CI and poor heart rate recovery at one minute post-exercise (HRR1) have been proposed as indices of autonomic imbalance [8].

To the best of our knowledge, little is known regarding autonomic nervous system functionality in patients with ILD and interstitial lung disease with pulmonary hypertension (ILD-PHTN). We believe exploring autonomic imbalance in these conditions may help us better elucidate the mechanism of exercise intolerance in patients with ILD and pulmonary hypertension. The objective of our study was to evaluate the presence of CI and abnormal HRR1 responses in patients with ILD and ILD-PHTN as measured during CPET.

## Materials and methods

Patient characteristics and study inclusion criteria

This was a retrospective study. The patients were included in the study if they had a diagnosis of interstitial lung disease alone (ILD group) or those with interstitial lung disease with pulmonary hypertension (ILD-PHTN group) and underwent CPET at our institution, Northwell Health, from 2008 to 2022. Interstitial lung disease was defined based on computed tomography (CT) scan consistent with the diagnosis and a restrictive ventilatory defect on pulmonary function test. Interstitial lung disease with pulmonary hypertension was defined using the above criteria for ILD along with a mean pulmonary artery pressure ≥ 20 mm of Hg and pulmonary capillary wedge pressure < 15 mmHg on right heart catheterization.

Our cohort study included a total of 32 patients. There were 21 patients with a diagnosis of ILD alone (ILD group) and 11 patients with interstitial lung disease with pulmonary hypertension (ILD-PHTN group). The ILD group had normal echocardiographic findings with normal ejection fraction and the ILD-PHTN group had evidence of pulmonary hypertension on the Echocardiogram. In fact we went a step further and made sure that the presence of pulmonary hypertension was confirmed on right heart catheterization.

Right heart catheterization

All patients with ILD-PHTN had their diagnosis confirmed on right heart catheterization. Hemodynamic parameters, including mean right atrial pressure (mRAP), mean pulmonary arterial pressure (mPAP), and pulmonary artery wedge pressure (PAWP) were recorded during this procedure. Cardiac output (CO) was determined utilizing the thermodilution method.

Pulmonary function test

Pulmonary function tests (PFT) were performed by experienced pulmonary function technicians using pneumographs (Vmax system, Viasys HealthCare, Conshohocken, PA, USA). Prebronchodilator and postbronchodilator responses were recorded as per the American Thoracic Society (ATS) guidelines [16]. Every subject completed at least three acceptable tests, with an interval of one minute. The variability between the three tests was less than 5%, and the results of the best trial were reported. Collected parameters included forced vital capacity (FVC), forced expiratory volume in one second (FEV1), FEV1/FVC, total lung capacity (TLC), residual volume (RV), RV/TLC and diffusing capacity of the lung for carbon monoxide (DLCO) (corrected for hemoglobin). PFT parameters were expressed as percentage of predicted values (%pred), which were calculated using the equations of Miller [17]. All patients had restriction ventilatory defects on PFT.

Cardiopulmonary exercise testing

Patients performed a symptom-limited incremental CPET, 5-15W/min. The CPET procedures adhered to the established guidelines set forth by the ATS and were executed utilizing a manually-braked bicycle, specifically the Lode Corival CPET ergometer from Sweden. Continuous monitoring of key parameters, including oxygen consumption (VO2) measured in milliliters per kilogram per minute (mL/kg/min), minute ventilation (VE) in liters per minute (L/min), and carbon dioxide output (CO2) in milliliters per minute (ml/min), was facilitated through the application of a CPX metabolic measurement cart (Vmax CPET system, Viasys HealthCare), equipped with rapid-response O2/CO2 analyzers.

Data were recorded as a mean of 10 seconds and VO2max was defined as the highest 30-second average of VO2. Heart rate was obtained from the R-R distance as established by an in-built 12 lead electrocardiogram. Blood pressure measurements were obtained at three-minute intervals through an automated cuff. Oxygen saturation (SpO2) levels were monitored via pulse oximetry.

The exercise protocol encompassed a three-minute resting phase, followed by three minutes of unloaded cycling maintained at 60 revolutions per minute. The rate of incremental workload adjustment during the test phase was tailored to the estimated exercise capacity of individual patients. The exercise session was terminated upon the patient's achievement of 85% of the age-predicted maximum heart rate, respiratory exchange ratio >1.04, or the occurrence of severe adverse events such as profound dyspnea, chest pain, or light‐headedness.

CPET data analysis

Age-predicted maximum heart rate (APMHR) was calculated using the Astrand equation (220 - age) [18], with the failure to attain 85% of APMHR during exercise indicating an impaired response. HRR1 was calculated as the difference between peak heart rate (peak HR) and HR one minute after exercise cessation. An HRR1 value < 16 was considered indicative of an abnormal autonomic response [13]. The Chronotropic Response Index (CRI) was employed to gauge the heart's responsiveness to physical activity, quantifying the change in heart rate relative to the increase in physical workload [6]. CRI was calculated as follows:

$$CRI=\frac{peak HR-resting HR}{(220-age)-resting HR}$$

In general, the term "chronotropic incompetence" is defined as the heart's inability to adequately increase its rate in response to physical activity or exercise [9]. For this study, CI was defined based on the following two criteria [19,20]: 1) Failure to attain 85% of the age-predicted maximum heart rate, and 2) A chronotropic response index (CRI) < 0.80 (80%). These criteria allowed us to discern and quantify the extent of chronotropic responsiveness in our study cohort, shedding light on the functional status of the cardiovascular system during exercise in this group of patients with interstitial lung disease and interstitial lung disease with pulmonary hypertension.

Statistical analysis

Continuous variables were presented as mean values along with their corresponding standard deviations, unless otherwise specified. Correlation between CRI and VO₂max was determined using Pearson’s correlation test. To discern differences between two groups, unpaired Student's T-tests and Fisher’s Exact Tests were employed. A p-value ≤ 0.05 was considered significant.

## Results

The ILD group (21 patients) consisted of 12 male and nine female patients, while the ILD-PHTN group (11 patients) consisted of 10 female patients and one male patient. The mean age of the ILD group was 60.33 ± 13.69 years and BMI was 26.42 ± 4.84, whereas the mean age of the ILD-PHTN group was 56.63 ± 14.19 years and BMI was 25.20 ± 4.58. Table 1 compares CPET parameters between the ILD group and the ILD-PHTN group.

**Table 1:** Cardiopulmonary exercise testing (CPET) parameters in interstitial lung disease (ILD) and ILD with pulmonary hypertension (ILD-PHTN) groups Values are presented as mean ± standard deviation. HR = heart rate, bpm = beats/minute, SBP = systolic blood pressure, HRR1 = heart rate recovery at one minute post-exercise, DLco = diffusing capacity of the lung for carbon monoxide, CRI = chronotropic response index, APMHR = age appropriate maximum heart rate, CI = chronotropic incompetence *Chronotropic incompetence was calculated as failure to achieve 85% of the age-predicted maximum heart rate and a chronotropic response index <0.8. **Heart rate recovery at 1-minute post-exercise (HRR1) was calculated as the difference between peak heart rate (peak HR) and HR one minute after exercise cessation. HRR1 value below 16 was considered abnormal.

	ILD (n=21)	ILD-PHTN (n=11)	p-value
VO₂max (ml/kg/min)	18.11 ± 4.48	14.15 ± 5.00	0.04
VeVCO₂	29.46 ± 4	33.51 ± 8	0.06
Maximum workload (W)	84.76 ± 36.10	49.82 ± 25.88	0.002
DLco%pred	60.33 ± 9.54	35.73 ± 15.70	0.0003
During rest			
HR rest (bpm)	92.90 ± 12.68	78.14 ± 15.53	0.014
SBP rest (mmHg)	120 ± 18	113 ± 17	0.12
During peak exercise			
HR peak (bpm)	132.90 ± 20.44	127.73 ± 19.90	0.25
SBP maximum (mmHg)	165.24 ± 27	143.55 ± 17	0.01
During recovery			
HR at 1 min of recovery (bpm)	115.14 ± 22.32	117.27 ± 16.25	0.38
SBP recovery (mmHg)	138.4 ± 24	126.2 ± 18	0.06
HRR1 (bpm)	18 ± 9	10 ± 7	0.015
CRI	0.691 ± 0.210	0.468 ± 0.179	0.005
CRI <0.8, n (%)	14 of 21 (66.66%)	11 of 11 (100%)	0.066
Fail to achieve 85% APMHR, n (%)	10 of 21 (47.62%)	10 of 11 (90.90%)	0.023
CI present*, n (%)	10 of 21 (47.62%)	10 of 11 (90.90%)	0.023
Abnormal HRR1** (<16bpm), n (%)	10 of 21 (47.62%)	9 of 11 (81.82%)	0.128
Both abnormal HRR1 and CI, n (%)	3 of 21 (14.28%)	8 of 11 (72.72%)	0.004

VO₂max

The mean VO₂max for the entire group was 16.75 ml/kg/min. There was a significant difference in VO₂max between the ILD and ILD-PHTN groups (18.11 ± 4.48 vs 14.15 ± 5.00, p < 0.05).

HRR1 in ILD and ILD-PHTN

The mean HRR1 for the entire group was 15.25 beats. There was a significant difference in HRR1 between the ILD and ILD-PHTN groups (18 ± 9 vs 10 ± 7, p < 0.05). Ten of 21 (47.62%) ILD patients had abnormal HRR1 < 16 beats compared to nine of 11 (81.82%) ILD-PHTN patients, however this difference was not statistically significant.

85% of age-appropriate maximum heart rate

Twenty of 32 patients (62.5%) failed to achieve 85% of their APMHR. In the ILD group, 10 of 21 patients (47.62%) did not reach this target, while in the ILD-PHTN group, 10 of 11 patients (90.90%) failed to attain 85% of APMHR (p < 0.05).

Chronotropic Response Index

**Figure 1:**
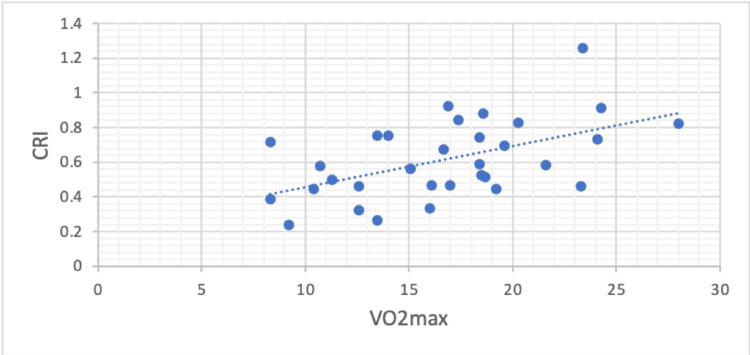
Scatterplot showing the relationship between VO2max and Chronotropic Response Index (CRI) in interstitial lung disease (ILD) and ILD with pulmonary hypertension (ILD-PHTN) patients. (Pearson correlation, r = 0.52, p =0.002)

VO₂max increased as the CRI increased (Pearson correlation, r = 0.52, p =0.002) (Figure 1). Twenty-five of all 32 patients (78.12%) demonstrated CRI < 0.8. Fourteen of 21 patients (66.66%) in the ILD group had CRI < 0.8, whereas all of the ILD-PHTN patients, 11 of 11 (100%), displayed CRI < 0.8 (p = 0.066).

Chronotropic incompetence

CI was diagnosed if there was a failure to reach 85% of APMHR with a CRI < 0.8. Twenty out of 32 patients (62.5%) had CI based on our criteria. There was a statistically significant difference in CI between patients with ILD-PHTN and in patients with ILD alone. Ten out of 11 (90.90%) in the ILD-PHTN group had CI compared to 10 out of 21 patients (47.62%) in the ILD group (p < 0.05; Fisher’s exact test).

In our study, eight of 11 (72.72%) ILD-PHTN patients had both an abnormal HRR1 and CI while three of 21 (14.28%) patients in the ILD alone group had both an abnormal HRR1 and CI (p < 0.05).

DLco%pred

The mean DLco%pred for all patients was 51.87. Both groups demonstrated reduction in DLco%pred but it was more pronounced in the ILD-PHTN group compared to the ILD group (35.73 ± 15.70 vs 60.33 ± 9.54, p < 0.05).

## Discussion

This study demonstrates that CI and poor HRR1 are present in patients with ILD and ILD-PHTN. The study also shows that CI and poor HRR1 seem to be more pronounced in patients with ILD-PHTN compared to patients with ILD alone (Table 1).

CI is most commonly identified when HR fails to reach a predefined threshold, typically set at either 85% or 80% of the APMHR achieved during an incremental dynamic exercise test [10]. The APMHR is classically described by the equation of Astrand et al. (APMHR = 220 - age) [18]. Although still widely accepted, the Astrand formula has been shown to overestimate maximum heart rate in younger people, while underestimating it in older people [21].

Alternatively, CI has also been determined through the change in heart rate from rest to the peak during an exercise test, commonly referred to as the HR reserve [10]. Since the proportion of HR achieved during exercise can be influenced by resting HR levels, evaluating the chronotropic response to exercise can also involve determining the fraction of HR attained at maximal effort. Consequently, an adjusted HR reserve is often calculated by the change in HR from rest to peak divided by the difference between resting HR and the age-predicted maximum heart rate which is also called the Chronotropic Response Index.

$$CRI=\frac{peak HR-resting HR}{(220-age)-resting HR}$$

This approach has been used by other authors in their studies adopting a criterion of CRI < 0.8 (80%) as an indicator of CI [10,19,20]. In our study, CI was defined based on both of the above-mentioned criteria I) Failure to attain 85% of the age-predicted maximum heart rate, and II) A CRI < 0.80 (80%). To the best of our knowledge CI is not well studied in patients with ILD alone and in patients with ILD-PHTN. In our study 20 patients out of total 32 patients (62.5%) had CI based on our above-mentioned criteria. CI appeared more common in the ILD-PHTN group (10 out of 11 patients (90.90%)) than in the ILD group (10 out of 21 patients (47.62%)).

In this study, we also measured the HRR1 in these individuals. While chronotropic incompetence focuses on the heart's ability to increase the heart rate during exercise, HRR1 measures the recovery phase, indicating how well the heart rate returns to baseline after exercise. Both CI and poor HRR1 are presumed to be indices of autonomic imbalance [8]. Autonomic dysfunction and its effects on symptomatology in patients with heart failure is well established [22]. However, its role in advanced lung disease has mainly been studied in chronic obstructive pulmonary disease (COPD) [6,8,23] and pulmonary hypertension patients [13,24].

The mechanism of dyspnea during exercise in ILD is multifactorial and related to myriad alterations of lung physiology which translate into a profound decrease in exercise capacity, and it is possible that underlying CI may be a contributing factor. Future studies are required to better delineate the role of CI in exercise intolerance in patients with ILD alone and those with patients with ILD-PHTN.

We need to point out that in our study, the ILD group's resting heart rate was higher than the resting heart rate of ILD-PHTN despite the ILD-PHTN group being sicker as measured by DLCO. This is due to the patients with ILD-PHTN being on beta blockers and some on calcium channel blockers which can affect the baseline heart rate. Despite this, the autonomic dysfunction as measured by CI and HRR1 was more pronounced in the ILD-PHTN group.

Mechanism of chronotropic incompetence in ILD

In normal subjects, heart rate is maintained by sympathovagal balance [25]. It has been established that both sympathetic activation and parasympathetic withdrawal work to increase the heart rate during exercise [24,26]. After exercise, sympathetic withdrawal and parasympathetic activation both contribute towards the recovery of the heart rate [27]. A slower heart rate recovery has been shown to mark an increased risk of mortality [14,15,28,29]. CI has also been shown to be an independent predictor of mortality in the general population [7,30]. We observed in our study that patients with ILD and ILD-PHTN have abnormal CI (an indicator of impaired sympathetic response) and HRR1 (indicating parasympathetic dysfunction), suggesting that both limbs of autonomic dysfunction are affected in this group of patients with advanced lung disease.

During exercise, heart rate regulation may show incompetence either as a delayed heart rate response, submaximal peak HR response or inadequate HR recovery [22]. In individuals with interstitial lung disease, a blunted heart rate response may occur possibly due to underlying autonomic dysfunction. The activity of autonomic nerves may be adversely affected due to persistent hypoxemia and increased respiratory effort that leads to sympathovagal imbalance. Hypoxemia in COPD has been shown to be associated with autonomic dysfunction [31] and similar hypoxemia-related sympathovagal imbalance mechanisms may be contributing to autonomic dysfunction in patients with ILD and ILD-PHTN.

Our study has some significant limitations, including retrospective design and small sample size, that could have affected the results. However, despite these limitations, the findings that CI and HRR1 are impaired with exercise are significant from clinical point of view for patients with ILD and ILD-PHTN. We chose HRR1 < 16 as a cutoff in our study as it has been suggested as a predictor of clinical deterioration in pulmonary hypertension [13]. We believe that CRI and HRR1 are simple, inexpensive, and easily collected parameters when subjects perform CPET and in future it may be possible that they can be used as patient-centered measures for disease progression in ILD and ILD-PHTN. Further studies are warranted to evaluate the effect of these parameters on patient prognosis and to examine whether pulmonary rehabilitation improves autonomic dysfunction in this group of patients.

## Conclusions

CI and poor HRR1 are observed in patients with ILD and ILD-PHTN. These parameters are more pronounced in patients with ILD-PHTN compared to patients with ILD alone. Since CI and HRR1 can easily be obtained during CPET, further studies need to explore if these parameters can find application as risk stratification tools for patients with ILD and ILD-PHTN.
